# An Investigation into High-Accuracy and Energy-Efficient Novel Capacitive MEMS for Tire Pressure Sensor Application

**DOI:** 10.3390/s24248037

**Published:** 2024-12-17

**Authors:** Liang Luo, Ziyuan Wang, Jianwei Chen, Aisn Gioronara Hui, Allwins Moore Rogikin, Rongzhen Liu, Yao Zhou, Zhujin Jiang, Changde He

**Affiliations:** 1 Department of Engineering Mechanics, Tsinghua University, Beijing 100084, China; medinathu@163.com; 2 School of Integrated Circuits, Southeast University, Nanjing 214135, China; 230228414@seu.edu.cn; 3 Shanghai Institute of Ceramics, Chinese Academy of Sciences, Shanghai 201800, China; chenjw@mail.sic.ac.cn; 4 Einsteck Inc., Palo Alto, CA 94305, USA; einsteck@126.com; 5 Golden Rogikin Limited, Kowloon 999077, Hong Kong; moore24@126.com; 6 School of Instrument and Electronics, North University of China, Taiyuan 030051, China; 13027020313@163.com; 7 School of Modern Post, Xi’an University of Posts and Telecommunications, Xi’an 710061, China; vejango123@163.com; 8 School of Mechanical and Electric Engineering, Soochow University, Suzhou 215021, China; zjjiang2024@suda.edu.cn

**Keywords:** MEMS, tire pressure monitoring system, capacitive, silicon–silicon bonding, ultra-low power consumption

## Abstract

Tire pressure monitoring systems (TPMSs) are essential for maintaining driving safety by continuously monitoring critical tire parameters, such as pressure and temperature, in real time during vehicle operation. Among these parameters, tire pressure is the most significant, necessitating the use of highly precise, cost-effective, and energy-efficient sensing technologies. With the rapid advancements in micro-electro-mechanical system (MEMS) technology, modern automotive sensing and monitoring systems increasingly rely on MEMS sensors due to their compact size, low cost, and low power consumption. This study presents a novel high-precision capacitive pressure sensor based on a capacitive micromachined ultrasonic transducer (CMUT) structure and a silicon–silicon direct bonding process. The proposed design offers exceptional performance with high accuracy, ultra-low power consumption, and reduced production costs, making it an optimal solution for enhancing the precision and efficiency of TPMS. Leveraging its low power requirements, capacitive sensing technology emerges as a superior choice for energy-efficient systems in the automotive industry.

## 1. Introduction

TPMSs are essential for real-time monitoring of tire temperature and pressure, offering timely warnings about air leaks, low pressure, or high temperatures to pre-emptively signal deteriorating tire conditions and ensure driving safety. Additionally, maintaining optimal tire pressure is crucial for prolonging tire life, minimizing wear on the suspension system, improving fuel economy and CO_2_ reduction [1,2,3]. This study introduces a micro tire pressure monitoring system characterized by ultra-low power consumption and high precision. The system aims to rectify the inadequate pressure measurement accuracy of existing sensor chips [4,5], enhancing measurement accuracy up to ±0.01 Bar (1 kPa) with a working current at the nanoampere level. This breakthrough is a direct result of advancements in integrated circuit (IC) technology and micro-electro-mechanical system (MEMS) technology, which allow for the production of highly precise micromechanical structures on a large scale [6,7]. The CMUT technology, driven by MEMS advancements, leverages semiconductor manufacturing processes to facilitate the mass production of transducers. This development guarantees the high reliability, uniformity, and cost-efficiency of the products, offering a solution for creating large-scale two-dimensional array devices for ultrasonic transducers [8,9].

This article introduces a cutting-edge, high-precision capacitive pressure sensing technology based on a CMUT structure. Utilizing the latest advancements in MEMS and wafer bonding techniques, the proposed scheme employs a capacitance detection-based pressure sensor [10]. The design incorporates an array cavity structure that provides precise and reliable tire pressure monitoring. The sensor stands out for its high measurement accuracy, robust stability, quick response time, and low power consumption, all packaged in a compact size with outstanding environmental adaptability. The theoretical accuracy of this system is pinpointed at ±0.01 Bar (1 kPa), while achieving ultra-low power consumption. Capacitive sensors, such as the one described here, are especially suited to low-power systems due to their power-efficient measurement principles, offering marked improvements in precision, energy efficiency, and noise reduction over resistive pressure sensors, which do not require a DC current bias. Notably, this capacitive tire pressure sensor is designed for up to 10 years of continuous operation, maintaining ultra-low power use throughout its extended service life [11,12].

## 2. Materials and Methods

**Figure 1:**
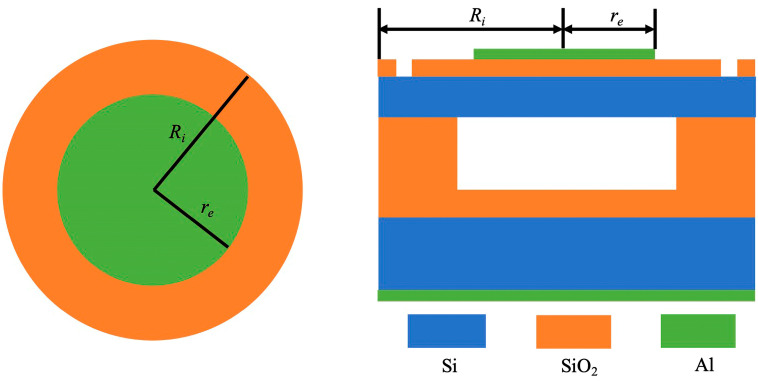
CMUT unit structure diagram.

The design of CMUT devices is heavily influenced by the choice of membrane shape, with common geometries including square, circular, and regular hexagonal. The anisotropic nature of silicon wafers in wet process technology usually results in square membrane structures, as this method typically produces square cavities. Despite the higher plot ratio appeal of regular hexagonal structures, both square and hexagonal films can lead to reduced manufacturing yields. This reduction is due to uneven stress distributions that occur during processing, which can affect device sensitivity. Square membranes, in particular, are susceptible to stress concentration at their sharp corners, potentially causing membrane rupture and thus yield loss. To mitigate these challenges, a circular thin-film structure has been proposed as a viable alternative [13,14,15,16]. The optimal CMUT unit, as structured in a top-down arrangement in Figure 1, includes an upper electrode, an insulation layer to prevent Ohmic contact, the core vibrating film, a cavity, peripheral support for structure and fixation, another insulation layer, a supportive substrate plane, and a lower electrode. $R_{i}$ is the radius of insulation layer, and re is the radius of top electrode. This design approach aims to minimize stress concentration, thereby enhancing device performance and production yield [7,16,17,18].

### 2.1. Parallel Plate Capacitance Model

**Figure 2:**
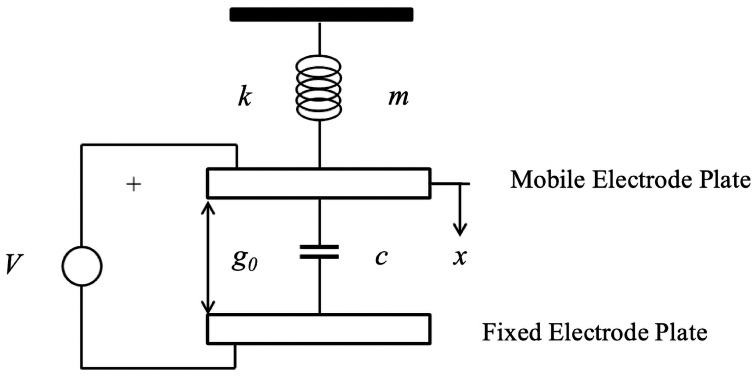
CMUT flat plate capacitor model.

CMUT can be seen as a parallel plate capacitor with a moving upper plate and a fixed lower plate. According to the unit structure and working principle of CMUT, the membrane can be seen as the upper moving plate, and CMUT can be equivalent to a parallel plate capacitance model composed of a mass block, spring, and parallel plate capacitance, as shown in Figure 2. *m* is the mass of the mass block; *k* is the spring elasticity coefficient; *V* is the DC bias voltage applied to the film and substrate; $g_{0}$ is the original cavity height, and *x* is the initial displacement of the film under DC bias voltage.

The force analysis of the upper plate can be obtained from Newton’s second law:

(1) $$F_{mass}+F_{e}+F_{s}=0$$

Among them, $F_{mass}$ represents the total external force applied to the thin film; $F_{e}$ represents the electrostatic force between two parallel plates caused by applied bias, and $F_{s}$ is the spring restoring force. The expression for electrostatic force $F_{e}$ is [17,19,20]

(2) $$F_{e}=-\frac{d}{d_{x}}\left(\frac{CV^{2}}{2}\right)=-\frac{1}{2}V^{2}\left[\frac{d}{dx}\left(\frac{\varepsilon_{0}A}{g_{0}-x}\right)\right]=\frac{\varepsilon_{0}AV^{2}}{2{\left(g_{0}-x\right)}^{2}}$$

where *A* is the equivalent area of the capacitor; $\varepsilon_{0}$ is the vacuum dielectric constant with a value of $8.854187817\times 10^{-12}\;F/m$. The spring restoring force $F_{s}$ can be expressed as

(3) $$\;F_{s}=-kx$$

### 2.2. Working Principle

**Figure 3:**
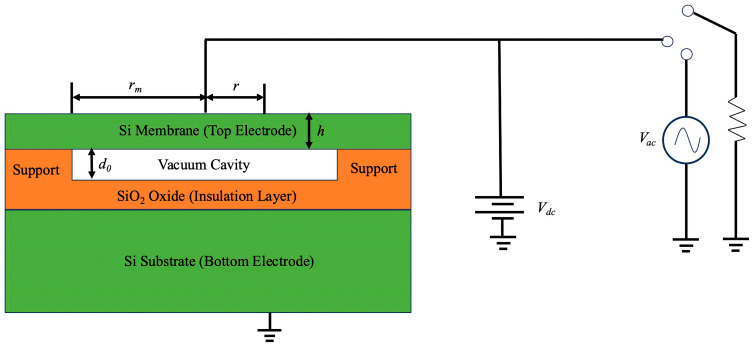
CMUT pressure unit structure.

The schematic diagram of the CMUT unit structure used for pressure measurement is as follows in Figure 3 [16,21]. The effective radius of the pressure silicon film is $r_{m}$; the thickness is h, and it is used as a pressure sensing element. The height of the vacuum chamber is $d_{0}$, and the silicon substrate is used as the bottom electrode of the CMUT. When external pressure is applied to the CMUT capacitive structure, the $d_{0}$ decreases, leading to an increase in capacitance. As a result, any applied pressure causes a corresponding change in capacitance.

The resonant characteristics of the pressure film in a capacitive pressure sensor based on the CMUT structure depend not only on its mechanical properties and geometric shape but also on the DC bias and measured pressure. When a DC bias voltage $V_{\mathrm{bias}}$ is applied, the membrane deflects towards the bottom plate, causing a shift in the resonant frequency of the membrane. Research has shown that within a lower bias voltage range, the resonant frequency varies linearly with pressure. If a bias voltage is applied to the electrode, the relationship between the resonant frequency and the pressure acting on the membrane under bias voltage is as follows:

(4) $$f_{p}=f_{0}\left(a-bP\right)$$

(5) $$f_{0}=\frac{0.83}{r_{m}}\sqrt{\frac{Eh^{3}}{m\left(1-v^{2}\right)}}$$

In Equation (4), $f_{p}$ is the resonant frequency under applied pressure *P*; $f_{0}$ is the natural frequency of the membrane, shown in Equation (5) [21,22], and *a* and *b* are two coefficients determined by the mechanical properties and geometric shape of the membrane, as well as the bias voltage $V_{\mathrm{bias}}$. *a* reflects the influence of DC bias voltage on resonant frequency, while *b* determines the resonant frequency shift caused by pressure *P* under bias voltage. The corresponding pressure sensitivity is defined as follows:

(6) $$S_{P}=\frac{1}{af_{0}}\frac{\partial f_{P}}{\partial P}=-\frac{b}{a}\left({\mathrm{ppmPa}}^{-1}\right)$$

Under the same external pressure, the membrane achieves a greater resonance frequency shift under DC bias. Compared with membranes without DC bias voltage, known as under bias voltage, pressure will cause greater changes in membrane offset and stress, which can be analyzed by the following two formulas [21,22]:

(7) $$\Delta w_{0}\left(V_{\mathrm{bias}\;},P\right)=\frac{\Delta P}{64\left[D-Q_{1}r_{m}^{4}/\left(42d_{0}\right)\right]}{\left(r_{m}^{2}-r^{2}\right)}^{2}$$

(8) $$\Delta w_{0}\left(P\right)=\frac{\Delta P}{64D}{\left(r_{m}^{2}-r^{2}\right)}^{2}$$

(9) $$Q_{1}=\frac{\varepsilon_{0}V_{\mathrm{bias}\;}^{2}}{2d_{0}^{2}}$$

(10) $$D=\frac{Eh^{3}}{12\left(1-v^{2}\right)}$$

$\Delta w_{0}$ is the deflection change in the clamping film; ΔP is the pressure change, and $Q_{1}$ represents the effect of the applied bias voltage $V_{\mathrm{bias}}$ in Equation (9) [21]. D represents the flexural stiffness of the CMUT membrane, which is often modeled as a thin plate. The stiffness D is given in Equation (10), where E is the Young’s modulus of the membrane material; h is the thickness of the membrane, and v is the Poisson’s ratio [21].

Equation (7) describes the change in membrane deflection caused by pressure when a bias voltage is applied, while Equation (8) represents the membrane deflection due only to measured pressure. As a result, DC-biased CMUTs achieve an amplified frequency shift, enhancing pressure sensitivity. Under identical pressure conditions, the biased membrane exhibits greater deflection than the unbiased membrane, resulting in a more pronounced frequency shift. Thus, DC-biased CMUTs can achieve amplified frequency shifts, improving pressure sensitivity [21,22,23,24,25,26,27,28].

### 2.3. Manufacturing Process

The fabrication of CMUTs hinges on two pivotal processing techniques. The first technique, known as the sacrificial layer release process, is the cornerstone of traditional CMUT manufacturing process. The second key process in CMUT production is wafer bonding technology, an approach refined alongside advancements in MEMS technology.

The two primary CMUT manufacturing processes each present distinct advantages. The sacrificial layer release process is well suited for initial stages of CMUT fabrication, while wafer bonding technology offers a more advanced, cleaner, and controlled approach for creating superior CMUT devices [7,18].

Traditional CMUT manufacturing relies on the sacrificial layer release method. Despite its widespread adoption, it has several limitations including a complex process flow, inconsistency in cavity size and distribution, and a high potential for contamination during production. However, as manufacturing technologies have evolved, wafer bonding has emerged as a preferred technique in recent years, addressing many of the issues associated with the sacrificial layer release method. Wafer bonding not only streamlines the fabrication process but also produces more uniformly sized cavities with reduced contamination, making it ideal for the production of large-area CMUT two-dimensional arrays. Consequently, wafer bonding has become the method of choice for CMUT two-dimensional array preparation [7,18].

**Figure 4:**
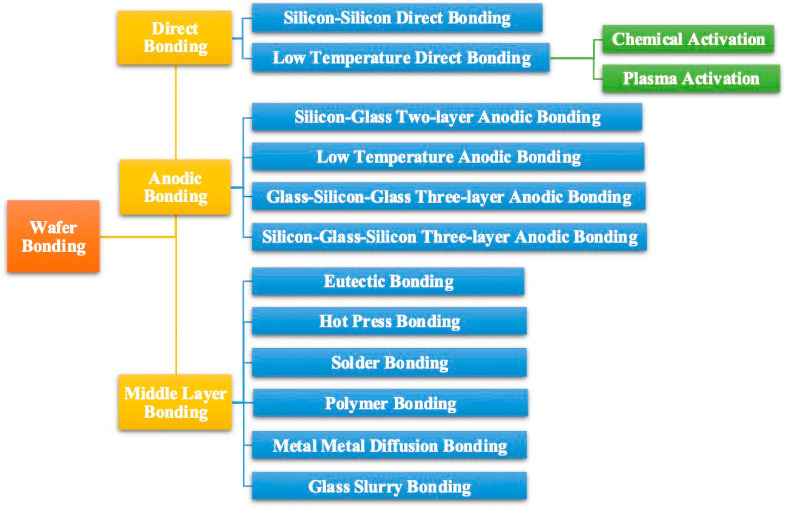
Classification of wafer bonding.

Currently, wafer bonding techniques can be classified into direct bonding, anodic bonding, and intermediate layer bonding as shown in Figure 4. Among these, low-temperature direct bonding is deemed most appropriate for CMUT two-dimensional array fabrication, as no intermediate layer is permissible when forming CMUT cavities. Options for bonding without an intermediate layer often necessitate chemical or plasma activation, which can complicate and hinder the bonding process. Thus, the simplicity and reliability of the silicon–silicon direct bonding process have made it the preferred method for assembling CMUT two-dimensional arrays [7,18,29,30,31].

In our design, we have implemented a low-temperature direct bonding process augmented by microcapacitor micro–nano technology. This approach has substantially increased the yield and consistency of microcapacitor structures and concurrently reduced the costs associated with manufacturing. For the CMUT pressure sensor assembly, silicon (Si) has been selected as the material for the pressure sensing membrane, silicon dioxide (SiO_2_) as the isolation and insulation layer, and aluminum (Al) as the electrode material [32,33,34].

**Figure 5:**
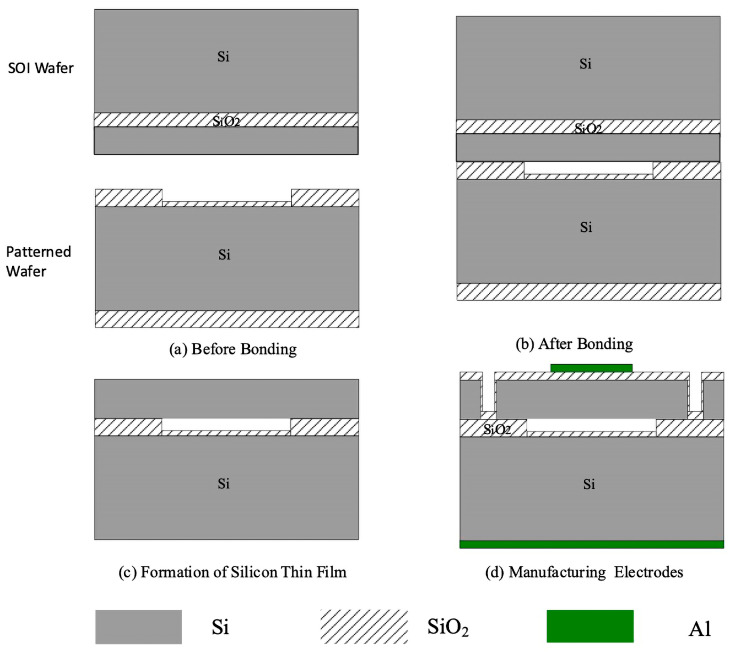
Schematic diagram of low-temperature direct wafer bonding manufacturing process.

The fabrication steps of the CMUT microcapacitor pressure sensing structure is delineated in Figure 5. First, a suitable SOI (Silicon-On-Insulator) wafer is selected, and a SiO_2_ (Silicon Dioxide) cavity is etched into the silicon, as shown in Figure 5a. Using a low-temperature direct bonding process, an SOI-bonded silicon wafer is formed, as depicted in Figure 5b. Next, the silicon layer and oxide layer of the SOI substrate are removed, resulting in a thin silicon film, as seen in Figure 5c. The insulation layer is oxidized; the top metal electrode is prepared; isolation trenches are etched, and the oxide layer on the back of the silicon is removed. Finally, the bottom electrode is fabricated to form an ohmic contact, completing the device structure, as illustrated in Figure 5d. This process leverages the uniformity of the SOI device layer, ensuring consistency in the sensor’s vibrating membrane structure.

The following are key requirements for low-temperature direct bonding: Surface Cleanliness: wafers must be free of contaminants for effective bonding; Surface Roughness: roughness should be smaller than 0.25 µm for hydrophobic wafers and no more than 0.5 µm for hydrophilic wafers; Total Thickness Variation (TTV): ideally around 2 µm; Surface Activation: required to improve bonding success. The bonding process has two stages: pre-bonding and high-temperature annealing. During pre-bonding, wafers are aligned and weakly bonded. High-temperature annealing then forms covalent bonds, ensuring a strong, permanent connection. Low-temperature direct bonding is used to bond the silicon wafer to the SOI, following these steps: Cleaning: both silicon and SOI wafers are cleaned with the RCA standard method to remove dust and residues that could hinder bonding; Surface Activation: the cleaned surfaces are treated with O_2_ plasma to enhance bonding success; Pre-Bonding: at room temperature, the silicon and SOI wafers are aligned and pre-bonded in a wafer bonder; High-Temperature Annealing: the pre-bonded wafers are then annealed at 1050 °C in an inert gas for 20 h, creating strong covalent bonds between the silicon and SOI. This process is essential for creating a stable, reliable bond between the silicon wafer and SOI, crucial for the structural integrity of the final device [32,33,34].

## 3. Results

### 3.1. COMSOL Multiphysics Simulation (COMSOL. Inc., Burlington, MA, USA)

**Figure 6:**
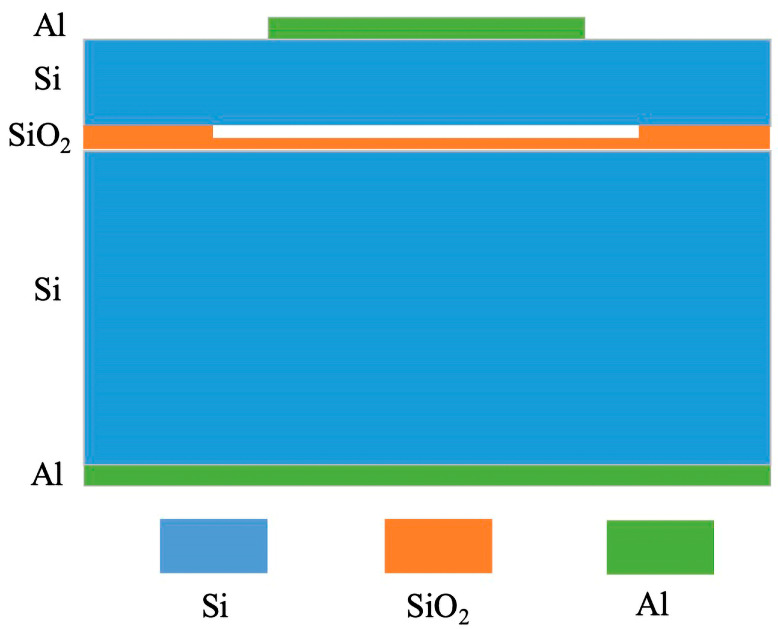
Design of CMUT pressure sensing unit structure.

The CMUT capacitive pressure sensor device design and parameters, which are shown in Figure 6 and Table 1, respectively, akin to other MEMS devices, encompass the integration of multiple physical field factors. To assess the sensor’s performance accurately, it is imperative to employ robust tools capable of coupling analysis between these physical fields. Such tools must meticulously delineate the interactions among the various physical fields to ensure a comprehensive understanding of the sensor’s operational dynamics. This detailed analysis is crucial for optimizing the sensor’s functionality and reliability in applications.

**Figure 7:**
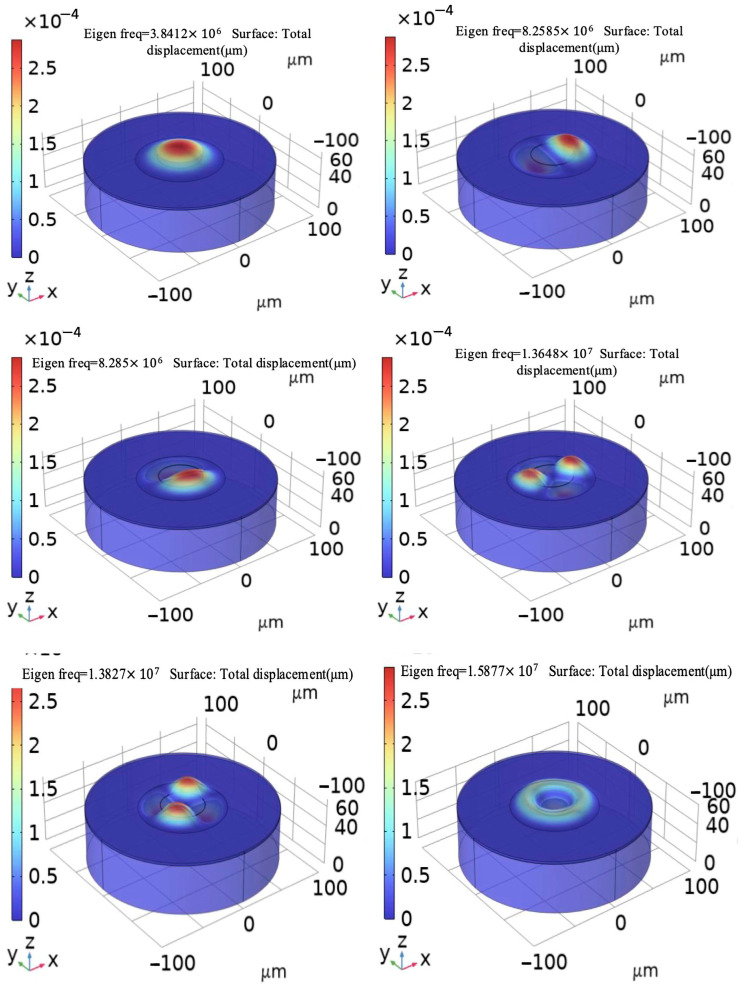
Analysis of the first six modes of CMUT pressure sensing unit structure.

Eigen frequency analysis is an essential step in understanding the displacement vibration characteristics of CMUT thin films and identifying their resonant frequencies. The resonant frequency serves as a critical parameter in evaluating the performance of a CMUT device. Through the construction of a three-dimensional model of a CMUT and examination of its characteristic frequencies, it is possible to discern the modal vibration modes and the resonant frequency. This information is pivotal as it directly influences the transducer’s efficiency and efficacy. A visualization of these modal patterns, typically the first six modes of vibration, where the modes and frequencies shown in Table 2, can be graphically represented and is often illustrated in technical figures to provide a clear understanding of the CMUT’s operational dynamics, as shown in Figure 7 [35,36,37,38,39].

By maintaining a constant membrane thickness and adjusting the membrane radius, we can analyze the resonance frequencies of a CMUT at various radii. This approach enables us to observe how changes in membrane size affect resonance frequency, providing valuable guidance for CMUT pressure sensor design optimization. By strategically adjusting membrane radius, we can strike an optimal balance between sensitivity and operational frequency, tailoring CMUT performance to meet specific needs.

### 3.2. Testing

**Figure 8:**
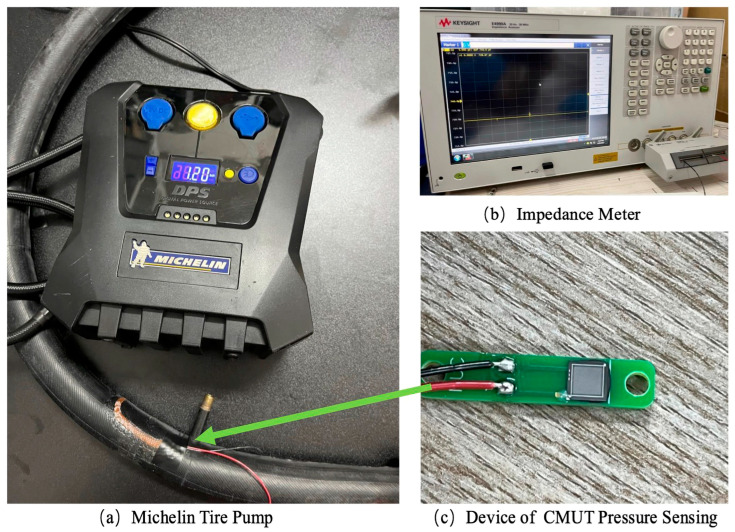
Photo of testing setup and CMUT pressure sensing device.

To evaluate the performance of the capacitive pressure sensor, the testing device shown in Figure 8c was installed within a bicycle tire in Figure 8a, which generally operates at pressures higher than those of automotive tires, thereby satisfying the pressure range requirements for automotive applications. Standard mountain bike tires are rated for pressures up to 2.8–4.5 Bar, while road bicycle tires can withstand pressures exceeding 6.8 Bar. The testing procedure began by creating a small opening in an intact, leak-free inner tube of a bicycle tire. The pressure sensor was then inserted into a small hole, and multiple layers of tire patch adhesive were then applied to ensure a secure and airtight seal around the sensor. The sensor leads were carefully extended through the opening for external connection. After allowing the patch adhesive to dry for 5–10 min, the inner tube was inflated using a Michelin tire pump (Michelin, Shanghai, China) equipped with both pressure control and display features in Figure 8a, facilitating accurate pressure monitoring during testing. This testing setup provides a robust and controlled method for assessing the pressure sensor’s accuracy and reliability under conditions analogous to those in automotive tire environments.

When measuring capacitance with a Keysight E4990A Impedance Analyzer (Keysight Technologies, Santa Rosa, CA, USA) as shown in Figure 8b, an AC voltage ranging from −20 V to +20 V is applied at a frequency of 1 kHz, which is far from the CMUT pressure sensing design resonant frequency. The testing data are listed in Table 3.

## 4. Discussion

In this CMUT pressure sensing array design, each unit features a pressure film with a thickness of 2 μm and a cavity height of 0.2 μm within a 30 × 30 array structure. The capacitive sensor’s nonlinear properties yield an average sensitivity of 154.6 aF/kPa, indicating that sensitivity fluctuates with pressure changes. At 230 kPa and 35.56 fF, maximum capacitance change is expected. At 1 kPa resolution, the calculation for the minimum charge variation for a single CMUT unit capacitor at 1.5V is detailed in the study.

(11) ΔQ=ΔC⋅U

The calculations reveal that the minimal change in capacitance due to pressure application on a CMUT device corresponds to a displacement of 1449 electrons across the capacitor plates, given the electron charge of $1.6\times 10^{-19}$ coulombs. To boost the system’s sensitivity, a matrix of sensor units can be created by connecting multiple capacitor units in parallel [40].

Moreover, CMUT technology offers benefits such as ease of mass production, circuit integration, structural robustness, and reliable operation in adverse conditions, making it exemplary for various applications.

Particularly for tire pressure monitoring sensors, the emphasis on power consumption is greater than that on accuracy. CMUT pressure sensitivity and precision meet measurement standards even without a bias voltage, significantly reducing power consumption. This low-power mode of operation enables the sensor to last up to 10 years with a low energy consumption, demonstrating CMUT potential for long-term, energy-efficient applications [40].

**Figure 9:**
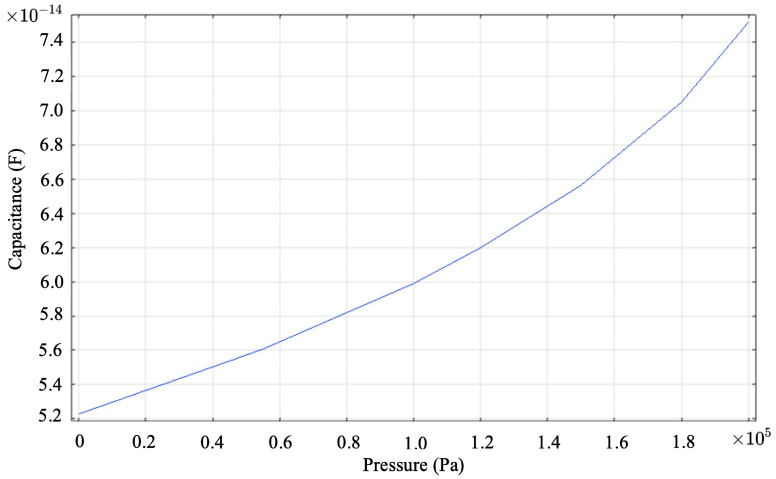
Simulation of capacitance and pressure relationship of CMUT pressure sensing structure.

**Figure 10:**
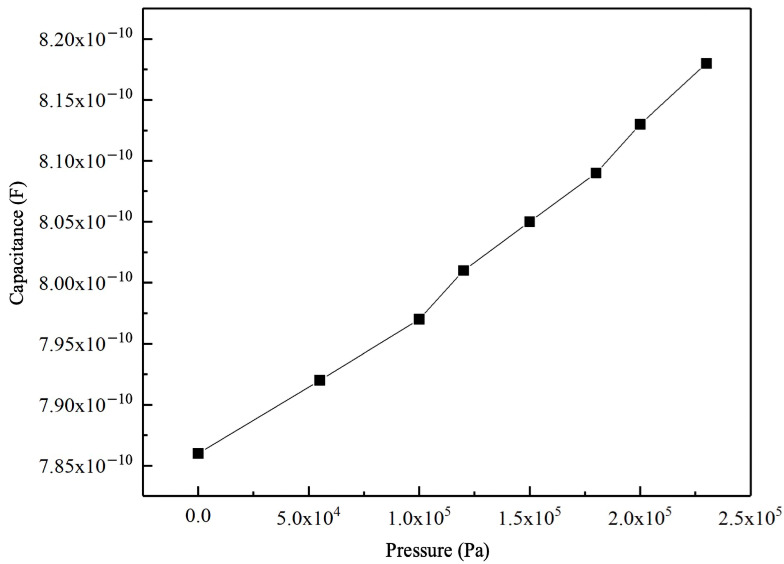
CMUT pressure device testing results showing relationship between capacitance and pressure.

The pressure measurement sensitivity of the simulated data for the CMUT-based pressure sensor designed in this study is 119 fF/kPa, with the result of the simulation shown in Figure 9, while the actual measurement sensitivity for tire pressure data is 139 fF/kPa, testing results are shown in Figure 10. The close match between the simulation and test data validates the design approach for the CMUT pressure sensor structure.

**Figure 11:**
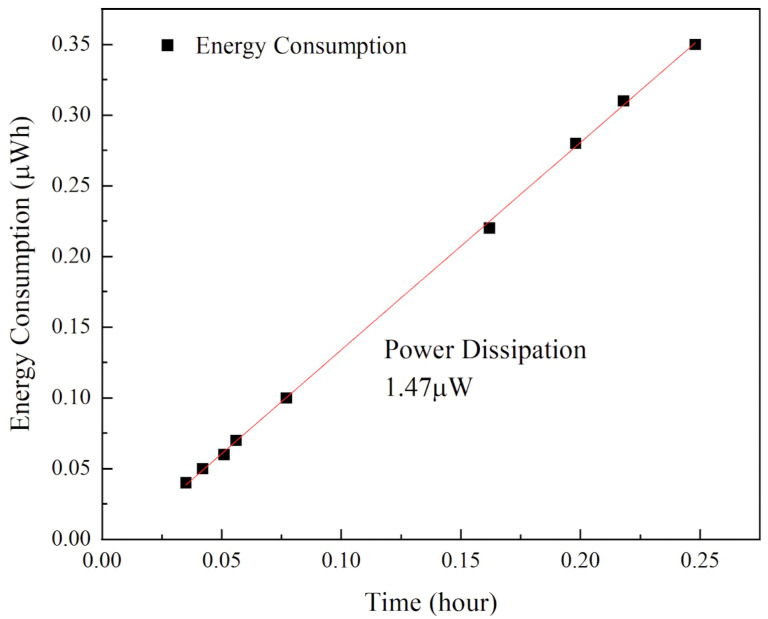
Power dissipation testing data of CMUT pressure device.

We utilized a CA-330PRO Dynamic Current Analyzer (Beijing Chipment Technology Co., Ltd., Beijing, China) to measure the power consumption of the CMUT pressure sensing device. The equipment specifications are as follows:

DC Voltage: from 0.0002 mV to 0.006 V;

DC Current: from 0.02 μA to 0.002 A;

Resistance: from 0.0002 Ω to 0.015 MΩ;

Figure 11 highlights the exceptionally low power consumption of the CMUT pressure sensing device. The measured power is 1.47 μW, achieved with a supply voltage of 9 V and a resulting current of 160 nA. This ultra-low power operation underscores the device’s suitability for applications where energy efficiency is critical.

The slope data presented in Table 4 show a clear dependence on the bias voltage. As the bias voltage increases, the slope values also grow larger. This relationship demonstrates that the slope is directly proportional to the pressure measurement sensitivity of the device. Higher bias voltages result in improved sensitivity, making the device more effective in detecting minute pressure changes.

**Figure 12:**
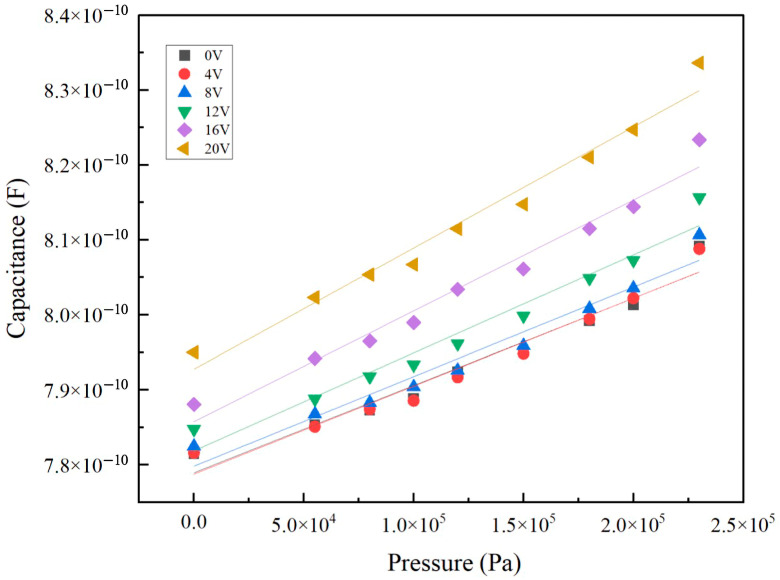
Capacitance changes with pressure of CMUT pressure sensing device at different DC bias.

Figure 12 further supports this observation by illustrating the capacitance variation in the CMUT pressure sensing device under different bias voltages. The results reveal that as the pressure changes, the capacitance becomes increasingly sensitive, particularly at higher bias voltages. This enhanced sensitivity is a direct consequence of the DC bias applied to the CMUT, which optimizes its operational characteristics for precise pressure measurement. The combination of the slope data and the capacitance behavior under varying bias voltages underscores the effectiveness of DC-biased CMUTs in enhancing pressure sensitivity. This makes them well suited for applications requiring high-precision and low-power pressure sensing, such as medical diagnostics, industrial monitoring, and other scenarios demanding real-time, ultra-sensitive pressure detection.

## 5. Conclusions

Cost-Effective manufacturing through low-temperature direct bonding: The CMUT pressure sensor is fabricated using silicon–silicon bonding technology, a method that combines high sensitivity with low manufacturing costs. This technique not only ensures robust structural integrity and excellent hermetic sealing but also simplifies the manufacturing process, making it suitable for large-scale production. The cost advantages of silicon–silicon bonding make CMUT sensors an economical choice for applications that require large sensor arrays, such as industrial pressure monitoring and consumer electronics. Versatile applications with ultra-low power consumption: The CMUT structure is highly versatile, functioning both as an ultrasonic transducer and a pressure sensor. This multifunctionality broadens its applicability across various fields, including medical ultrasound imaging and pressure monitoring. Furthermore, its ultra-low power consumption makes it ideal for devices with limited energy resources, such as tire pressure monitoring systems (TPMSs), where battery life is critical. The CMUT’s low power requirements make it a valuable component in the next generation of smart, autonomous systems that prioritize energy efficiency. Integration potential in IoT and automotive systems: With its high sensitivity, low power consumption, and cost-effective production, the CMUT pressure sensor is well suited for integration into IoT-enabled and automotive systems. In automotive TPMS, for example, CMUT sensors provide real-time pressure data with minimal power use, enhancing safety without imposing additional strain on the vehicle’s electrical system. In IoT applications, CMUT-based pressure sensors deliver precise, reliable data for smart home systems, environmental monitoring, and industrial automation, contributing to more intelligent and responsive systems. With its advanced silicon fabrication, cost efficiency, and versatile design, CMUT technology offers a powerful solution for high-performance, affordable, and energy-efficient sensing applications.

## Figures and Tables

**Table 1:** Design parameters of CMUT pressure structure sample.

Structural Parameters	Size
Membrane Radius (μm)	46
Membrane Thickness (μm)	2
Electrode Radius (μm)	23
Electrode Thickness (μm)	0.5
Cavity Height (μm)	0.2

**Table 2:** The first six modes and frequencies of CMUT unit structure.

Mode	Eigen Frequency/MHz	Mode	Eigen Frequency/MHz
First Mode	3.8412	Fourth Mode	13.648
Second Mode	8.2585	Fifth Mode	13.827
Third Mode	8.285	Sixth Mode	15.877

**Table 3:** Test data of CMUT pressure sensing structure for tire pressure measurement.

Pressure (Pa)	Capacitance (F)
0	7.8578 × 10^−10^
55,000	7.9174 × 10^−10^
100,000	7.973 × 10^−10^
120,000	8.008 × 10^−10^
150,000	8.0452 × 10^−10^
180,000	8.0917 × 10^−10^
200,000	8.13093 × 10^−10^
230,000	8.1833 × 10^−10^

**Table 4:** DC bias effect of the CMUT pressure sensing device.

DC Bias (V)	Slope
0	1.16367 × 10^−16^
4	1.17213 × 10^−16^
8	1.194 × 10^−16^
12	1.30619 × 10^−16^
16	1.47752 × 10^−16^
20	1.6166 × 10^−16^

## Data Availability

Data are contained within the article.
